# Outcomes and prognostic factors of alternative treatment regimens for angioimmunoblastic T-cell lymphoma: a retrospective analysis

**DOI:** 10.3389/fonc.2025.1585013

**Published:** 2025-09-10

**Authors:** Jiaqin Yan, Jiebing Wang, Junhui Zhang, Xudong Zhang, Lei Zhang, Zunmin Zhu, Mingzhi Zhang

**Affiliations:** ^1^ Department of Oncology, The First Affiliated Hospital of Zhengzhou University, Zhengzhou, Henan, China; ^2^ Department of Otolaryngology, The Third Affiliated Hospital of Zhengzhou University, Zhengzhou, Henan, China; ^3^ Hematology Department of Henan Provincial People’s Hospital, Zhengzhou, Henan, China

**Keywords:** angioimmunoblastic T cell lymphoma, response rate, progression-free survival, overall survival, prognostic factor

## Abstract

**Background:**

Angioimmunoblastic T-cell lymphoma (AITL), representing the second most prevalent subtype of peripheral T-cell lymphoma, currently lacks standardized frontline therapeutic strategies.

**Methods:**

In this study, we evaluated the survival outcomes and prognostic factors in 154 patients with AITL treated with one of four regimens: CHOP (cyclophosphamide, vincristine, epirubicin, prednisone), CHOPE (CHOP + etoposide), CPET (chidamide, prednisone, etoposide, thalidomide), or GDPT (gemcitabine, cisplatin, dexamethasone, thalidomide). Among them, 144 patients had complete survival follow-up data. Survival differences across groups were analyzed using the log-rank test, while variations in clinical parameters were assessed via chi-square tests and one-way ANOVA. Univariate and multivariate Cox regression analyses were conducted to identify factors associated with progression-free survival (PFS) and overall survival (OS).

**Results:**

The 5-year OS and PFS rates for the entire cohort were 36.6% (95% CI: 0.275–0.488) and 32.2% (95% CI: 0.233–0.451), respectively. Patients who were younger (<60 or <70 years), had Ann Arbor stage I/II disease, or exhibited lower Eastern Cooperative Oncology Group (ECOG) performance status scores demonstrated significantly improved OS and PFS following treatment. Notably, among patients with ECOG <2, those treated with the CPET regimen achieved longer PFS and OS compared to those receiving CHOP or CHOPE. In contrast, for patients with ECOG ≥2, no significant survival differences were observed across treatment regimens. Both univariate and multivariate analyses identified ECOG performance status as an independent prognostic factor for survival outcomes.

**Conclusion:**

For patients with a low ECOG performance status, the CPET regimen may offer promising survival outcomes.

## 1 Introduction

Angioimmunoblastic T-cell lymphoma (AITL), a distinct subtype of peripheral T-cell lymphoma (PTCL), is characterized by unique clinicopathological and genetic features. Representing approximately 1-2% of all non-Hodgkin lymphomas and 15-20% of PTCL cases (1), this aggressive lymphoid malignancy primarily affects elderly patients, with a median diagnostic age of 65 years. Characteristic clinical manifestations include B symptoms, generalized lymphadenopathy, hepatosplenomegaly, anemia, and hypergammaglobulinemia (2). Histopathological hallmarks consist of clonal T-cell infiltration, aberrant follicular dendritic cell proliferation, and prominent high endothelial venules. Molecular analyses have established the follicular T-helper (Tfh) cell as the cellular origin of AITL, leading to its classification as a PTCL subgroup with TFH phenotype in the revised 2016 World Health Organization (WHO) classification (3).

Patients diagnosed with AITL generally have poor outcomes. A large international retrospective study of 282 patients, enrolled between 2006 and 2018, reported 5-year overall survival (OS) and progression-free survival (PFS) estimates of 44% and 32%, respectively (4). However, there remains no clear consensus on the optimal frontline management of AITL. Most clinical practice guidelines recommend initiating treatment through therapeutic clinical trials or with regimens such as CHOP (cyclophosphamide, vincristine, doxorubicin, prednisone) or CHOPE (cyclophosphamide, vincristine, doxorubicin, prednisone, etoposide) (5, 6). The Nordic Lymphoma Group (NLG) reported 5-year OS and PFS rates of 52% and 49%, respectively, for AITL patients treated with either CHOP or CHOEP in a large prospective Phase II trial (7). Recent clinical efforts have also focused on promising targeted therapies aimed at improving the prognosis of AITL (8–10).

The prognostic factors of AIT have been extensively studied in many researches. Most of these models are based on patients’ clinical parameters, although some models also incorporate gene expression characteristics (11). The International Prognostic Index (IPI) scoring system incorporates five parameters: age > 60 years, performance status (PS) ≥ 2, more than one extranodal site (ENS), B symptoms, and a platelet count below 150 × 10^9^/L. This model demonstrates superior performance in predicting the 5-year survival rate of AITL patients compared to other models (12). Recent research has introduced a new risk stratification tool for AITL patients, incorporating age, CRP levels, ECOG performance status, and β2-microglobulin. This scoring system demonstrated superior discriminatory power over conventional prognostic models, effectively categorizing patients into three distinct risk tiers with 5-year overall survival rates of 63% (low-risk), 54% (intermediate-risk), and 21% (high-risk) (4). However, numerous uncertainties remain regarding the prognostic factors of AITL, necessitating larger sample sizes and additional cohort studies for further investigation.

In the present study, we analyzed a cohort of retrospectively enrolled patients from two hospitals in China. We report the outcomes of patients treated with different regimens and evaluated prognostic factors.

## 2 Materials and methods

### 2.1 Patients

A total of 154 newly diagnosed, untreated patients with AITL were enrolled from two centers: the First Affiliated Hospital of Zhengzhou University and Henan Provincial People’s Hospital. Inclusion criteria were:(1) pathologically confirmed diagnosis of AITL according to the World Health Organization (WHO) classification; (2) availability of complete clinical data, including baseline staging information, treatment regimens, and response evaluation;(3) receipt of at least one line of therapy. Exclusion criteria were: (1) comorbid malignancies; (2) severe dysfunction of vital organs; (3) incomplete follow-up data;(4) active severe infections or uncontrolled immune-mediated diseases. Disease staging was defined using the Ann Arbor staging system. Bone marrow biopsy was performed on all patients as part of the diagnostic work-up. All procedures involving human subjects adhered to the ethical standards of the institutions and were conducted in accordance with the 1964 Helsinki Declaration and its subsequent amendments, or comparable ethical standards. Patient data, including age, gender, date of diagnosis, Ann Arbor stage, ECOG performance status (Grade 0: Fully active, able to perform all pre-disease activities without restriction. Grade 1: Restricted in physically strenuous activity but ambulatory and capable of light work. Grade 2: Ambulatory and capable of self-care but unable to perform work activities. Up and about >50% of waking hours. Grade 3: Limited self-care, confined to bed/chair >50% of waking hours. Grade 4: Completely disabled; unable to perform any self-care; totally confined to bed/chair. Grade 5: Death), Prognostic Index for T-cell lymphoma (PIT) score (Age >60 years, Elevated serum LDH (above institutional upper limit of normal) Poor performance status (ECOG ≥2), Bone marrow involvement), presence of B symptoms, bone marrow involvement, the number of extranodal areas involved, and laboratory parameters such as albumin, globulin, lactate dehydrogenase (LDH), β2-microglobulin (β-MG), and others, were collected from hospital records at the time of diagnosis.

### 2.2 Chemotherapy regimens

A total of 58 patients (37.6%) received the CHOP regimen, 33 patients (21.3%) received the CHOPE regimen, 35 patients (22.6%) were treated with the CPET regimen, 28 patients (19.1%) received the GDPT regimen. The dosing schedule, timing of administration, and route of delivery for each therapeutic protocol are listed in **Supplementary Table 2**.

### 2.3 Efficacy evaluation and follow-up

Treatment response was assessed using imaging modalities (PET-CT or CT) and classified into four categories according to Lugano 2014 criteria: complete remission (CR), partial remission (PR), stable disease (SD), and progressive disease (PD). The overall response rate (ORR), defined as the combined CR and PR rates, was evaluated after every two chemotherapy cycles. Patient outcomes were determined through telephone interviews or medical record reviews, with follow-up data collected until May 19, 2023. Ten patients were lost to follow-up due to unavailable contact information (CHOP: n=3; CHOPE: n=2; CPET: n=2; GDPT: n=3); their detailed information is presented **in Supplementary Table 1**. Follow-up duration was calculated from diagnosis to either death or last contact. PFS was measured from diagnosis to disease progression, death from any cause, or last follow-up, while OS was defined as the time from diagnosis to death from any cause or last follow-up.

### 2.4 Statistical analysis

All statistical analyses were conducted using SPSS (version 27.0) and R (version 4.0.1) software packages. Continuous variables were analyzed using one-way ANOVA, whereas categorical variables were compared using chi-square tests. Survival outcomes, including overall survival (OS) and progression-free survival (PFS), were evaluated by Kaplan-Meier analysis with between-group differences determined through log-rank testing. To identify significant prognostic factors, we performed both univariate and multivariate analyses using Cox proportional hazards regression models. Statistical significance was defined as a two-sided p-value < 0.05.

## 3 Results

### 3.1 Clinical features of all patients

The cohort comprised 154 patients with a male predominance (99 patients, 64.3%) and a median age at diagnosis of 62 years (range: 26–89 years). Key clinical features at presentation included: B symptoms in 70 patients (51.3%), hyperglobulinemia in 45 (29.2%), elevated LDH in 98 (65.3%), and bone marrow involvement in 51 (33.1%). Most patients (89.0%, n=137) presented with advanced-stage disease (Ann Arbor stage III/IV), while 25 (16.6%) had involvement of >2 extranodal sites. As shown in **Table 1**, these baseline characteristics were well-balanced across treatment groups except for ECOG performance status. Notably, the proportion of patients with ECOG ≥2 differed significantly among groups (*p*=0.019): CHOP (51.7%, n=30), CHOPE (27.0%, n=20), CPET (21.6%, n=16), and GDPT (22.9%, n=8).

**Table 1:** The clinical features of patients receiving different regimens.

Characteristics	total	CHOP	CHOPE	CPET	GDPT	P value
Number of patients	154	58(37.6%)	33(21.4%)	35(22.7%)	28(18.2 %)	
Gender						0.236
male%	99(64.3%)	33(56.9%)	20(60.6%)	27(77.1%)	19(67.9%)	
female(%)	55(35.7%)	25(43.1%)	13(39.4%)	8(22.9%)	9(32.1%)	
Age	62(26-89)	62(38-83)	56(43-77)	62(38-89)	63(26-73)	0.738
ECOG≥2(%)	74(48.1%)	30(51.7%)	8(10.8%)	20(27.0%)	16(21.6%)	0.019
PIT(%)						0.107
0-2	95(62.9%)	36(62.1%)	26(78.8%)	21(60.0%)	12(48.0%)	
3-4	56(37.1%)	22(37.9%)	7(21.2%)	14(40.0%)	13(52.0%)	
B symptoms(%)	70(51.3%)	35(60.3%)	13(39.4%)	18(51.4%)	13(46.4%)	0.257
Ann arbor staging						0.735
I-II	17(11.0%)	7(12.1%)	2(6.1%)	5(14.3)	3(10.7%)	
III-IV	137(89.0%)	58(87.9%)	31(93.9%)	30(85.7%)	24(89.3%)	
Anemia	59(38.3%)	21(36.2%)	16(48.5%)	13(37.1%)	9(32.1)	0.567
WBC	6.6(4.1-9.4)	6.6(4.6-10.1)	7.3(3.4-9.4)	5.4(3.4-7.7)	6.8(4.3-10.4)	0.238
PLT	193(132-250)	190(133-250)	197(115-249)	186(116-232)	206(134-258)	0.810
Albumin decreased	80(52.6%)	31(54.4%)	20(60.6%)	16(45.7%)	13(48.1%)	0.615
Globulin elevated	45(29.2%)	13(22.4%)	13(39.4%)	7(20.0%)	12(42.9%)	0.075
LDH elevated(%)	98(65.3%)	38(66.7%)	22(68.8%)	19(55.9%)	19(70.4%)	0.603
CRP elevated(%)	92(76.7%)	35(85.4%)	21(72.4%)	21(70.0%)	15(75.0%)	0.423
PCT elevated(%)	75(89.3%)	28(93.3%)	19(79.2%)	16(94.1%)	12(92.3%)	0.305
β2-MG elevated(%)	63(52.9%)	23(54.8%)	13(61.9%)	15(48.4%)	12(48.0%)	0.744
Bone marrow involvement(%)	51(33.1%)	19(32.8%)	11(33.3%)	11(31.4%)	10(35.7%)	0.987
Involved extranodal site≥2(%)	25(16.6%)	9(15.8%)	5(15.8%)	8(22.9%)	3(11.1%)	0.653
CR(%)	20(14.7%)	8(15.1%)	6(22.2%)	3(9.4%)	3(12.5%)	0.564
ORR(%)	52(38.2%)	16(30.2%)	12(44.4%)	16(50.0%)	8(33.3%)	0.203

### 3.2 Comparison of response rates among four treatment regimens

The overall cohort (n=154) demonstrated a CR rate of 14.7% and an ORR of 38.2%. When analyzed by treatment regimen, CR rates were 15.1% for CHOP, 22.2% for CHOPE, 9.4% for CPET, and 12.5% for GDPT (*p*=0.564), with corresponding ORR rates of 38.2%, 30.2%, 44.4%, and 50.0%, respectively (*p*=0.203) (**Table 1**). Notably, patients receiving CHOP or CHOPE regimens exhibited higher myelosuppression rates compared to other treatment groups (**Table 2**). Given the baseline imbalance in ECOG performance status among groups, we performed subgroup analyses stratified by ECOG score (<2 vs ≥2). However, neither CR nor ORR showed statistically significant differences among the four treatment regimens in either ECOG subgroup (**Table 3**).

**Table 2:** Response rate of four groups in ECOG<2 and ECOG≥2 subgroups.

Response	CHOP	CHOPE	CPET	GDPT	p value
ECOG<2					
CR(%)	3(21.4%)	3(33.3%)	2(13.3%)	1(11.1%)	0.589
ORR(%)	5(35.7%)	4(44.4%)	10(66.7%)	4(44.4%)	0.390
ECOG≥2					
CR(%)	5(12.8%)	3(16.7%)	1(5.9%)	2(13.3%)	0.804
ORR(%)	11(28.2%)	8(44.4%)	6(35.3%)	4(26.7%)	0.617

**Table 3:** Adverse event statistics across different treatment regimens.

Adverse events	CHOP(58)	CHOPE(33)	CPET(55)	GDPT(28)	p value
Myelosuppression	38	26	8	9	<0.001
Severe hematologic adverse reactions	9	5	3	3	0.79
Gastrointestinal reactions	7	5	4	3	0.958
Hepatic dysfunction	3	0	0	3	0.101
Cardiotoxicity	4	2	0	0	0.26
Venous thrombosis	0	1	3	4	0.011
Skin rash	0	0	3	2	0.029

### 3.3 Comparison of prognosis among four treatment regimens

**Figure 1:**
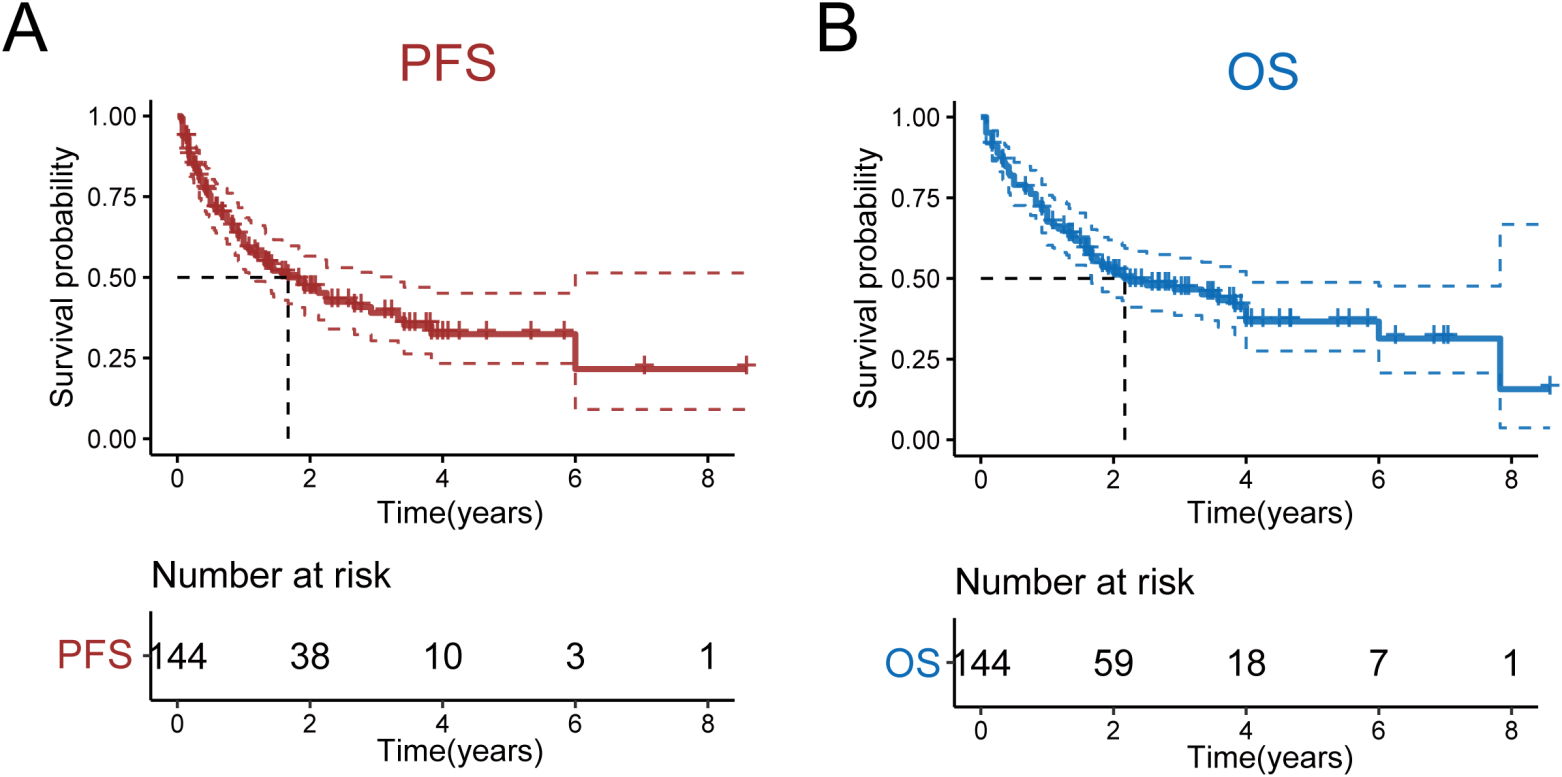
OS and PFS of all patients. **(A)** OS of all patients. **(B)** PFS of all patients.

**Figure 2:**
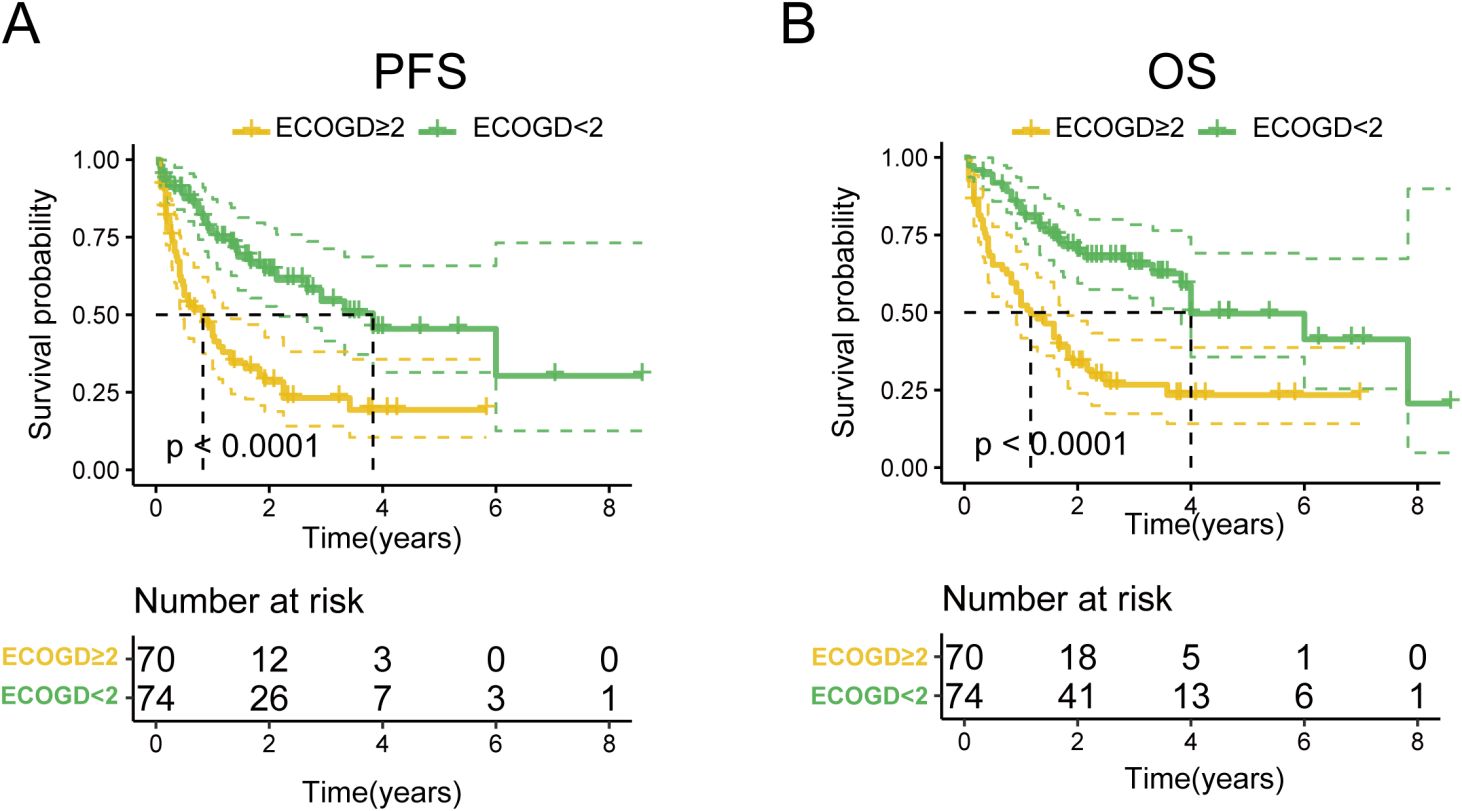
OS and PFS of patients in the ECOG < 2 and ECOG ≥ 2 groups. **(A)** OS of patients in the ECOG < 2 group. **(B)** PFS of patients in the ECOG ≥ 2 group.

As of May 2023, the median follow-up duration for all patients was 19 months, with a range from 1 to 103 months. The 5-year PFS and OS rates for all patients were 32.2% (95% CI: 0.233-0.451) and 36.6% (95% CI: 0.275-0.488), respectively. The median PFS and OS were 1.67 and 4 years, respectively (**Figures 1A, B**). Notably, no statistically significant differences were observed in either OS or PFS among patients receiving the four treatment regimens(**Supplementary Figures 1A, B**). Subsequent stratification by pathological parameters revealed significantly superior OS and PFS in patients aged <60/70 years compared to their ≥60/70 counterparts (**Supplementary Figures 2A, B**). Concurrently, patients with Ann Arbor stage I/II disease demonstrated significantly superior OS and PFS compared to advanced-stage counterparts (**Supplementary Figures 3A, B**).We also scored patients based on the PIT score and found that those with lower PIT scores had longer PFS and OS after treatment(**Supplementary Figures 4A, B**). Patients stratified by ECOG performance status (1/2/3) demonstrated a significant inverse correlation between ECOG score and survival outcomes, revealing that superior OS and PFS with lower ECOG scores (**Figures 2A, B**; **Supplementary Figures 5A, B**). Univariate and multivariate analyses revealed that an ECOG score of ≥ 2 was a significant factor for both PFS (*p* = 0.010) and OS (*p* = 0.042) (**Table 4**).

**Table 4:** Prognostic parameters analysis.

Characteristics	OS				PFS			
	Univariate analysis		Multivariate analysis		Univariate analysis		Multivariate analysis	
	HR,95% CI	p value	HR,95% CI	p value	HR,95% CI	p value	HR,95% CI	p value
gender	0.80(0.50-1.29)	0.355			0.77(0.48-1.23)	0.27		
age>60	2.40(1.46-3.95)	<0.001			1.96(1.20-3.19)	0.007		
ECOG≥2	2.77(1.73-4.43)	<0.001	2.35(1.03-5.37)	0.042	2.68(1.68-4.28)	<0.001	2.98(1.30-6.88)	0.010
B symptom	1.53(0.97-2.41)	0.068			1.44(0.92-2.27)	0.115		
Ann arbor(III-IV)	3.30(1.04-10.47)	0.043			3.88(1.22-12.31)	0.021		
anemia	0.84(0.53-1.33)	0.457			0.95(0.60-1.50)	0.819		
PIT≥3	2.26(1.43-3.55)	<0.001			2.18(1.39-3.43)	<0.001		
albumin decreased	1.81(1.14-2.86)	0.012			1.94(1.21-3.09)	0.005		
globumin elevated	1.64(1.03-2.61)	0.038			1.50(0.94-2.40)	0.087		
LDH elevated	1.40(0.86-2.23)	0.179			1.70(1.04-2.78)	0.035		
PCT elevated	1.16(0.41-3.22)	0.783			1.23(0.44-3.44)	0.691		
CRP elevated	4.96(1.98-12.42)	<0.001			5.25(2.10-13.18)	<0.001		
β-MG elevated	2.60(1.48-4.55)	<0.001			2.12(1.21-3.74)	0.009		
bone marrow involvement	0.59(0.74-1.19)	0.59			0.86(0.54-1.36)	0.518		
involved extranodal areas≥2	1.25(0.70-2.25)	0.45			1.28(0.72-2.29)	0.404		

**Figure 3:**
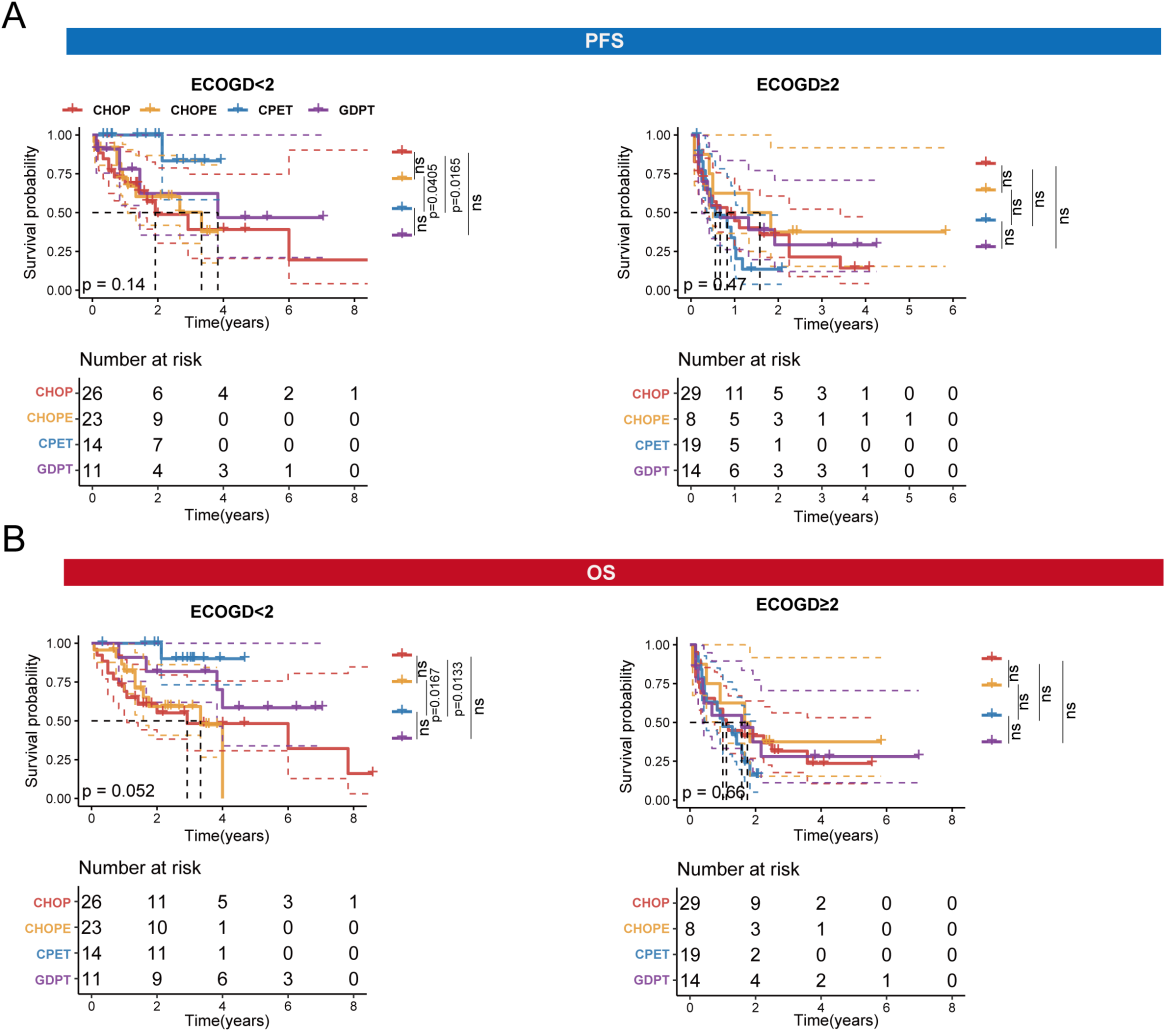
OS and PFS of four treatment regimens in the two subgroups: ECOG < 2 and ECOG ≥ 2. **(A)**. PFS of four treatment regimens in the ECOG < 2 and ECOG ≥ 2 subgroups. **(B)** OS of four treatment regimens in the ECOG < 2 and ECOG ≥ 2 subgroups.

Next, we compared the PFS and OS among patients treated with the four different regimens. In the ECOG < 2 subgroup, we found that patients treated with the CPET regimen had significantly longer PFS compared to those treated with the CHOP (*p* = 0.0165) and CHOPE (*p* = 0.0405) regimens. Similarly, patients treated with the CPET regimen had longer OS compared to those treated with the CHOP (*p* = 0.0133) and CHOPE (*p* = 0.0167) regimens. However, for patients with an ECOG performance status of ≥ 2, no significant differences in PFS or OS were observed between the four treatment regimens (**Figures 3A, B**).

## 4 Discussion

This study evaluated 154 patients with AITL treated with four regimens (CHOP, CHOPE, CPET, or GDPT), enabling comparative analysis of therapeutic outcomes in this WHO-classified lymphoma subtype. Through this approach, we assessed OS and PFS differences across treatment modalities while accounting for prognostic factors.

Currently, no standard chemotherapy regimen exists for newly diagnosed AITL. Although retrospective studies suggest limited efficacy of anthracycline-based approaches, CHOP remains the preferred treatment option (13). While the addition of etoposide to CHOP (CHOPE) may induce deeper responses in PTCL, this benefit comes with increased toxicity (14). Notably, a meta-analysis found no significant differences in treatment outcomes (including CR, PR, or ORR) between CHOP and CHOPE regimens for PTCL patients (15).In the latest clinical trial, the CHOPE regimen demonstrated higher CR (complete response) and ORR (overall response rate) of 72.7% and 81.8%, respectively, compared to the CHOP regimen (42.4% and 63.6% (16).However, in our study results, both treatment regimens demonstrated significantly lower CR and ORR compared to those reported in this trial, with no statistically significant difference observed between the two regimens.

Molecular studies have established that AITL pathogenesis involves characteristic mutations (RHOA, TET2, DNMT3A, and IDH2) (17, 18), all subject to acetylation regulation. As epigenetic modulators, histone deacetylase inhibitors like chidamide demonstrate therapeutic efficacy in AITL by modulating both histone and non-histone protein acetylation (19, 20). A Phase II trial of Chinese patients (n=71) reported superior response rates with CPET (ORR: 90.2%; CR: 54.9%) (10) compared to our retrospective data (ORR: 66.7%; CR: 13.3%). Notably, both studies showed comparable 2-year survival outcomes (PFS: 66.5% vs 82%; OS: 82.2% vs 89%), with our observed differences potentially attributable to smaller sample size, missing data, and confounding variables. Similarly, while a prospective trial demonstrated GDPT’s superiority over CHOP in 4-year outcomes (PFS: 63.6% vs 53.0%, *p*=0.035; OS: 66.8% vs 53.6%, *p*=0.039), our analysis revealed no significant survival differences between regimens.

Current literature reports conflicting findings regarding prognostic factors for AITL (4, 21), including age, ECOG performance status, Ann Arbor stage, and laboratory parameters (LDH, CRP, β-MG). Our multivariable analysis identified ECOG status as the sole significant predictor for both OS and PFS. While the PIT score remains an established prognostic tool for PTCL, it demonstrated no significant predictive value for either OS or PFS in our cohort. This discrepancy may reflect our study’s limited sample size or the therapeutic heterogeneity across treatment regimens.

This study has several limitations. First, the relatively small sample size precluded more extensive subgroup analyses. Second, missing follow-up data from some patients may have introduced potential bias. Third, although all patients received the same treatment protocol, the inclusion of subjects from two different medical centers might have led to potential environmental bias.

Our analysis stratified patients by ECOG performance status (ECOG <2 vs ≥2), revealing differential treatment responses. Notably, patients with ECOG <2 demonstrated significantly improved survival outcomes with CPET and GDPT regimens compared to CHOP/CHOPE. In contrast, ECOG ≥2 patients showed comparable survival across all regimens, suggesting equivalent efficacy in this less chemotherapy-tolerant population. These findings underscore the prognostic value of ECOG status in treatment selection, where robust patients (ECOG <2) may benefit from more intensive therapies while frail patients (ECOG ≥2) could potentially receive less aggressive regimens without compromising outcomes. However, the limited sample size in the ECOG ≥2 subgroup (n=42) warrants larger prospective studies to validate these observations and optimize therapeutic strategies for AITL.

This finding holds promise for optimizing clinical practice by: 1) preventing overtreatment in patients with ECOG≥2, thereby reducing treatment-related toxicity; and 2) guiding ECOG<2 patients toward more effective CPET/GDPT regimens to improve overall therapeutic outcomes in AITL. Further multicenter real-world studies are warranted to validate its generalizability.

## 5 Conclusion

In this retrospective study, we evaluated the prognostic impact of four commonly used treatment regimens in patients with AITL. Our findings suggest that, among patients with an ECOG performance status of < 2, treatment with the CPET regimen is associated with improved PFS and OS.

## Data Availability

The original contributions presented in the study are included in the article/supplementary material. Further inquiries can be directed to the corresponding authors.

## Supplementary material

Supplementary Figure 1 OS and PFS outcomes across four treatment regimens. **(A)**. PFS of patients received four treatment regimens. **(B)**. OS of patients received four treatment regimens.

Supplementary Figure 2 Age-stratified analysis of OS and PFS in patients with AITL. **(A)**.PFS and OS in patients aged ≥60 versus <60 groups. **(B)**. PFS and OS and PFS in patients aged ≥70 versus <70 groups.

Supplementary Figure 3 Ann arbor stage-stratified analysis of OS and PFS in patients with AITL. **(A)**. PFS in patients with Ann arbor stage I-II versus III-IV. **(B)**. OS in patients with Ann arbor stage I-II versus III-IV.

Supplementary Figure 4 PIT score-stratified analysis of OS and PFS in patients with AITL. (A). PFS and OS in patients with PIT score ≥2 versus ECOG < 2. **(B)**. PFS and OS in patients with PIT score ≥3 versus ECOG < 3.

Supplementary Figure 5 ECOG-stratified analysis of OS and PFS in patients with AITL. **(A)**. PFS and OS in patients with ECOG ≥1 versus ECOG < 1. **(B)**. PFS and OS in patients with ECOG ≥3 versus ECOG < 3.
