# Supplementary figures and images for: Outcomes and prognostic factors of alternative treatment regimens for angioimmunoblastic T-cell lymphoma: a retrospective analysis
Source: Front Oncol. 2025 Sep 10;15:1585013. doi: 10.3389/fonc.2025.1585013 (PMC12457117; doi:10.3389/fonc.2025.1585013)

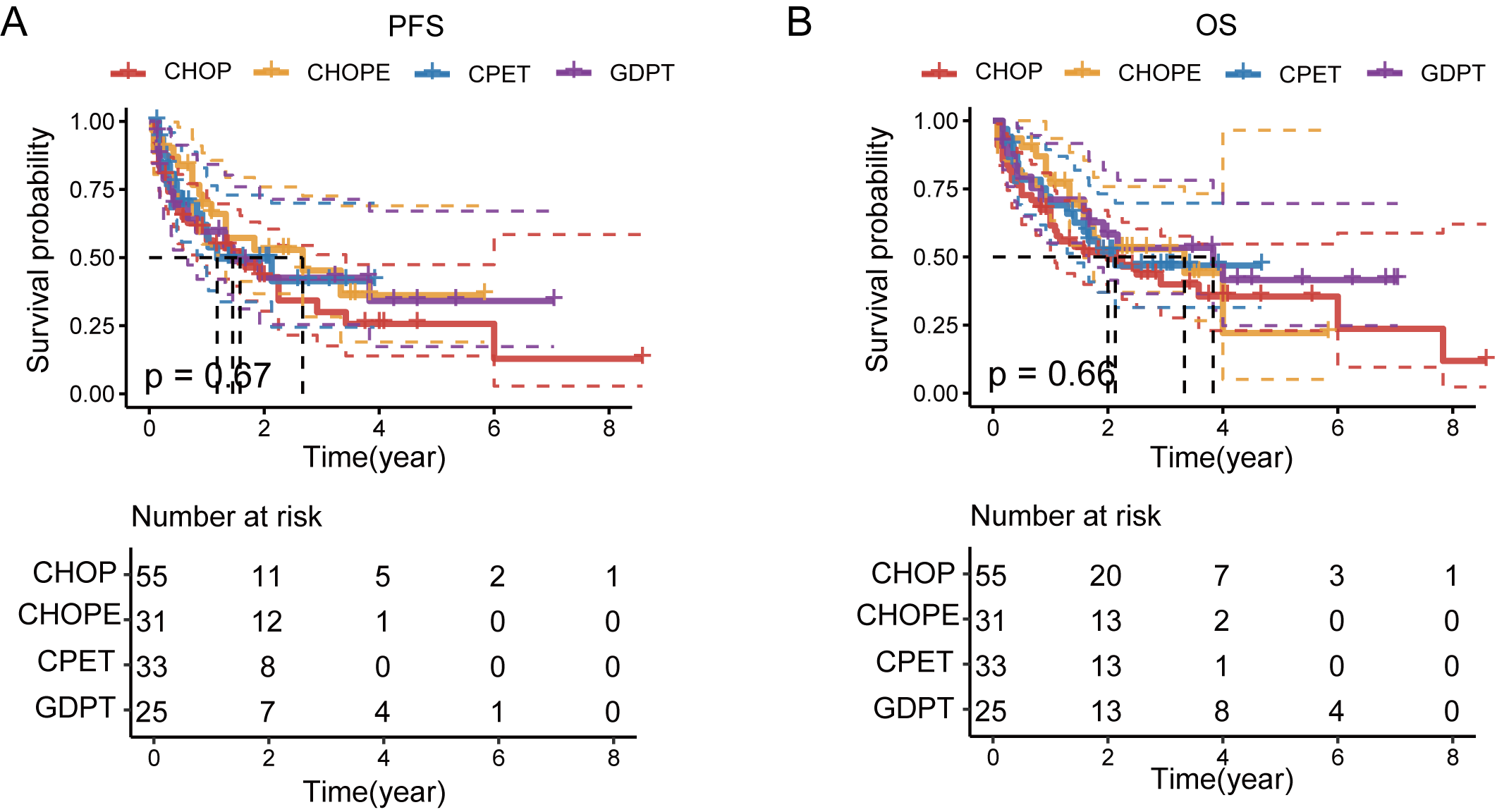

Supplement: Supplementary Figure 1 — OS and PFS outcomes across four treatment regimens. (A). PFS of patients received four treatment regimens. (B). OS of patients received four treatment regimens. [file Image1.tif]

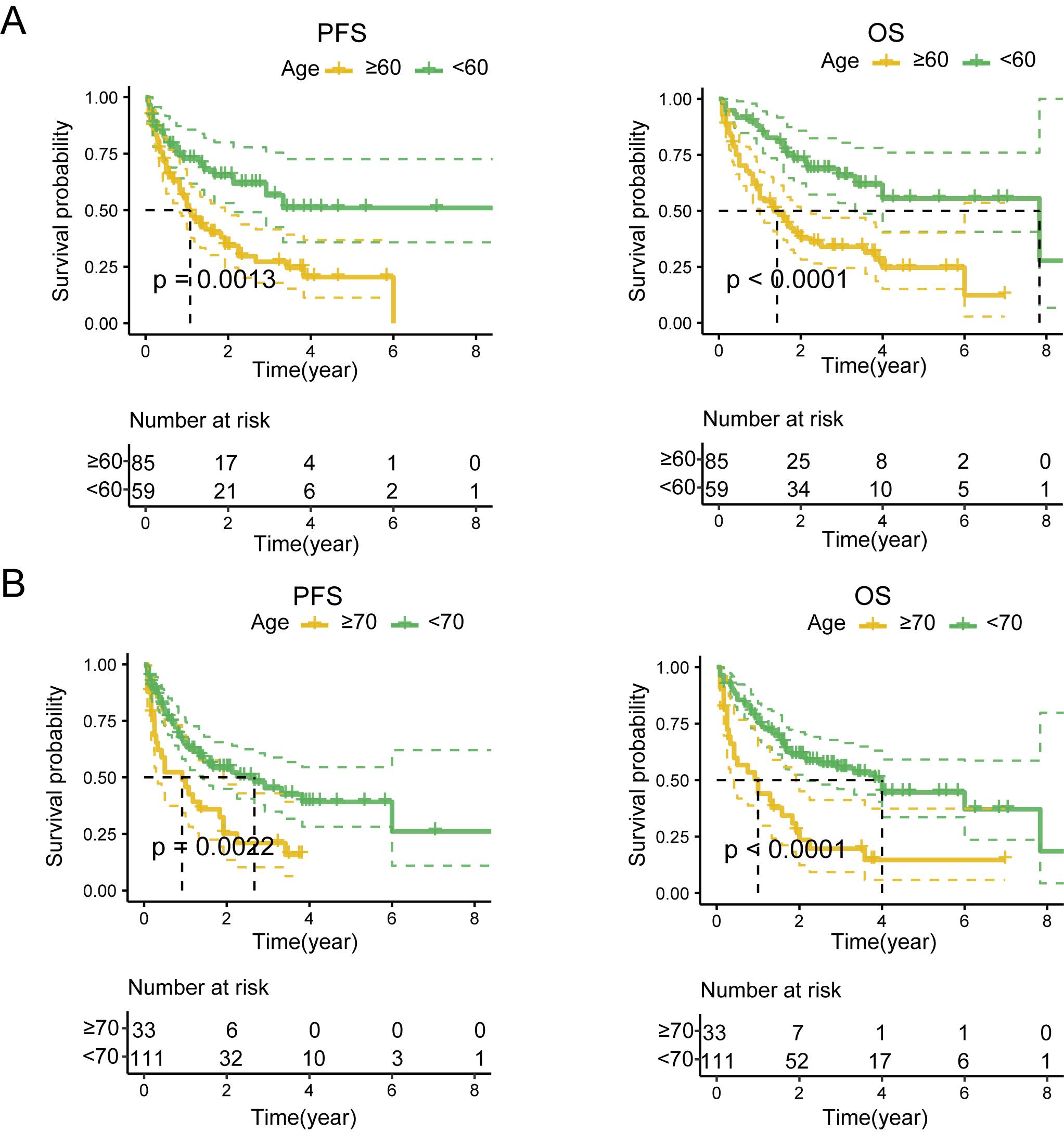

Supplement: Supplementary Figure 2 — Age-stratified analysis of OS and PFS in patients with AITL. (A).PFS and OS in patients aged ≥60 versus <60 groups. (B). PFS and OS and PFS in patients aged ≥70 versus <70 groups. [file Image2.tif]

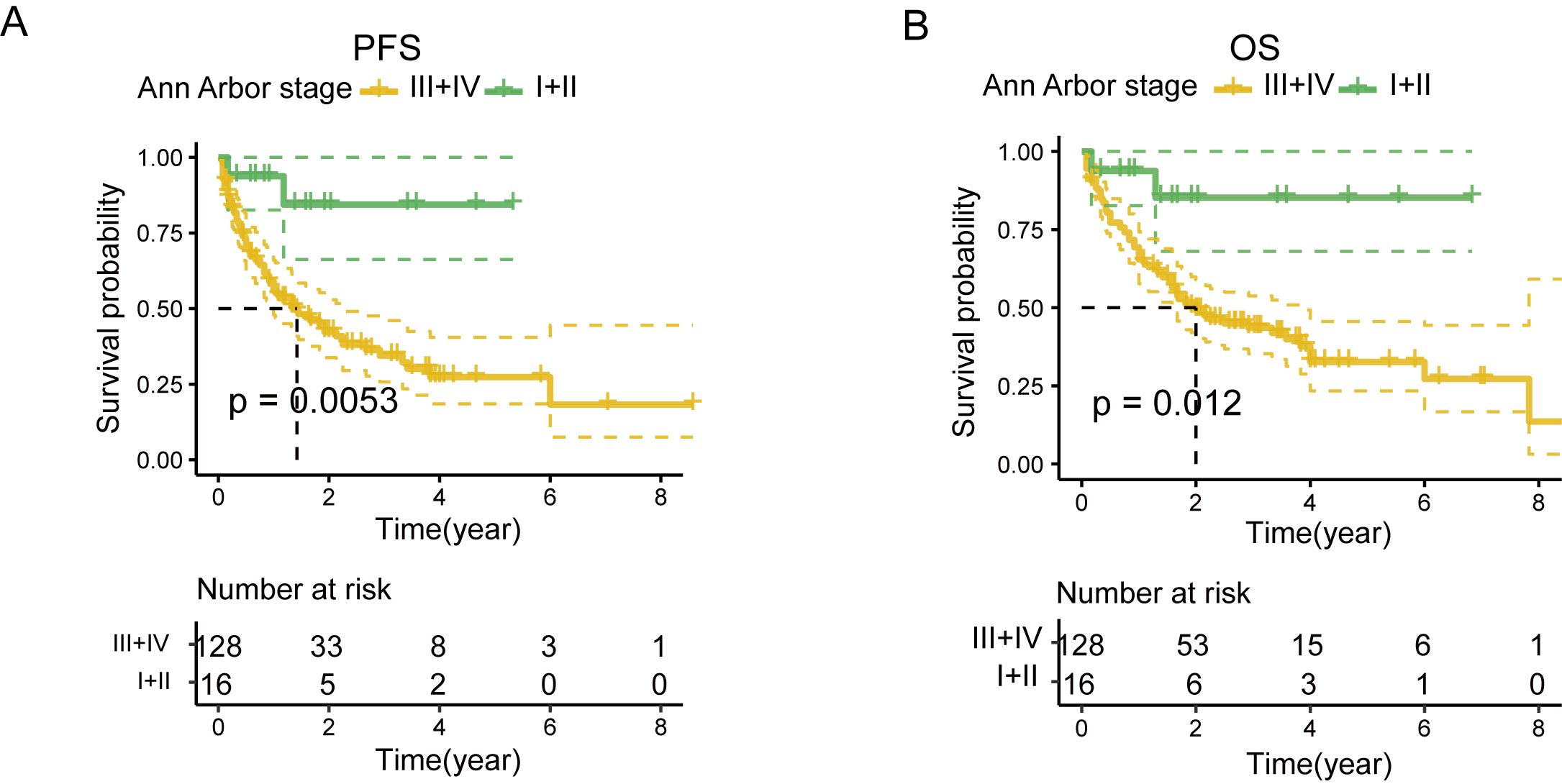

Supplement: Supplementary Figure 3 — Ann arbor stage-stratified analysis of OS and PFS in patients with AITL. (A). PFS in patients with Ann arbor stage I-II versus III-IV. (B). OS in patients with Ann arbor stage I-II versus III-IV. [file Image3.tif]

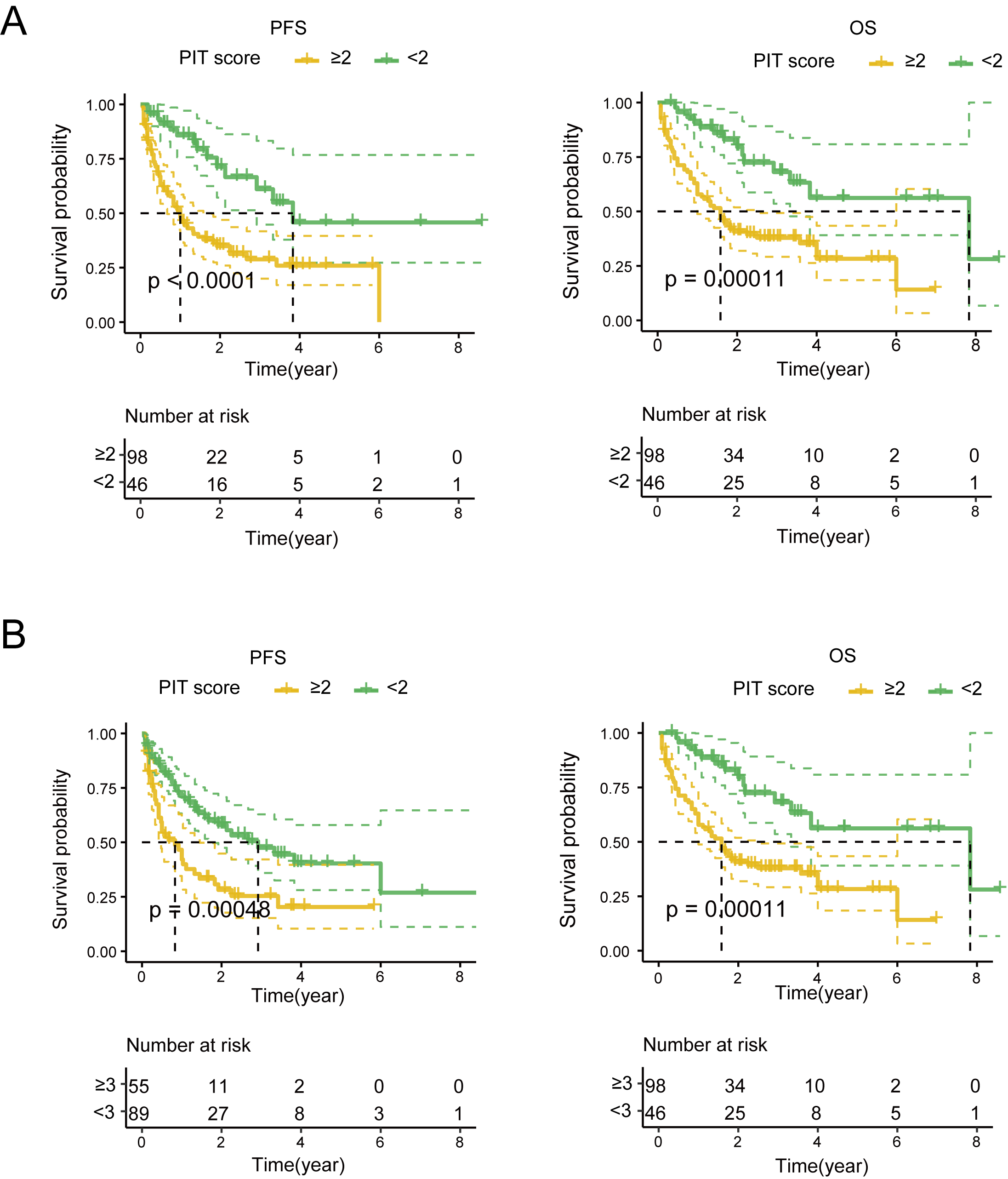

Supplement: Supplementary Figure 4 — PIT score-stratified analysis of OS and PFS in patients with AITL. (A). PFS and OS in patients with PIT score ≥2 versus ECOG < 2. (B). PFS and OS in patients with PIT score ≥3 versus ECOG < 3. [file Image4.tif]

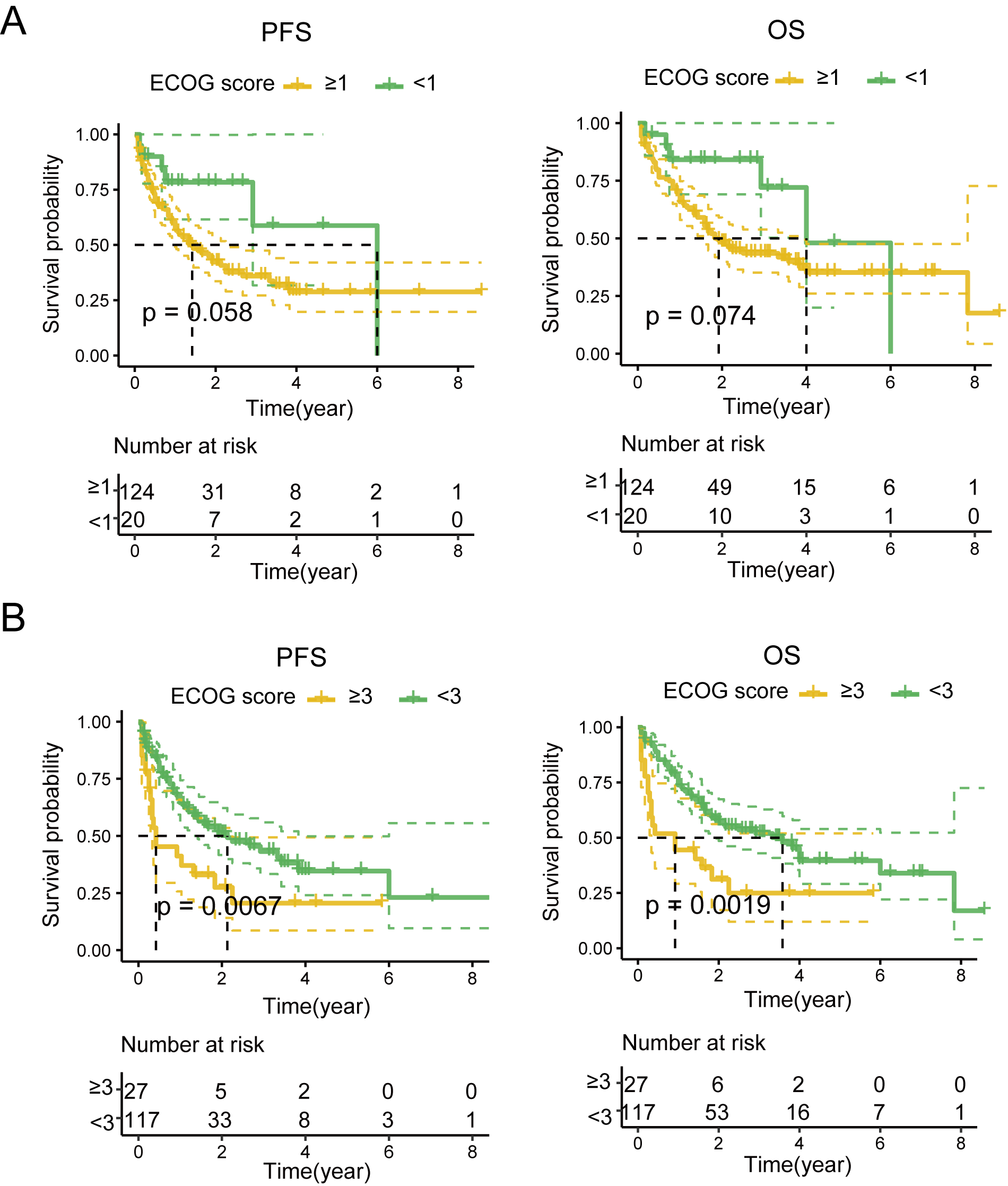

Supplement: Supplementary Figure 5 — ECOG-stratified analysis of OS and PFS in patients with AITL. (A). PFS and OS in patients with ECOG ≥1 versus ECOG < 1. (B). PFS and OS in patients with ECOG ≥3 versus ECOG < 3. [file Image5.tif]
