# Supplementary material for: Outcomes and prognostic factors of alternative treatment regimens for angioimmunoblastic T-cell lymphoma: a retrospective analysis
Source: Front Oncol. 2025 Sep 10;15:1585013. doi: 10.3389/fonc.2025.1585013 (PMC12457117; doi:10.3389/fonc.2025.1585013)
Supplement: Supplementary file 7 [file Table2.docx]

| Treatment regimen | Drugs | **Route of administration** | **Dosage** | Administration time |
| --- | --- | --- | --- | --- |
| CHOP | **C**yclophosphamide | IV | 750mg/m^2^ | d1 |
|  | Vincristine | IV | 1.4mg/m^2^ | d1 |
|  | **Epirubicin** | IV | 60mg/m^2^ | d1 |
|  | **Prednisone** | PO | 100mg/m^2^ | d1-5 |
| CHOPE | **C**yclophosphamide | IV | 750mg/m^2^ | d1 |
|  | Vincristine | IV | 1.4mg/m^2^ | d1 |
|  | **Epirubicin** | IV | 60mg/m^2^ | d1 |
|  | **Prednisone** | PO | 100mg | d1-5 |
|  | **Etoposide** | Ivgtt | 100mg | d1-3 |
| CPET | **Chidamide** | PO | 30mg | twice weekly |
|  | **Etoposide Capsules** | PO | 100mg | d1-d5 |
|  | **Prednisone** | PO | 100mg | d1-5 |
|  | **Thalidomide** | PO | 100mg | Daily |
| GDPT | Gemcitabine | Ivgtt | 0.8 g/m² | d1-8 |
|  | Cisplatin | Ivgtt | 75mg/m^2^ | d1-3 |
|  | **Dexamethasone** | Ivgtt | 20mg/m^2^ | d1-5 |
|  | **Thalidomide** | PO | 100mg | Daily |

SupplementaryTable2. Dosage and administration details of treatment regimens

Abbreviations

PO:per os; IV:intravenous; Ivgtt:intravenous guttae; d:day
